# High prevalence of hyperglycaemia and the impact of high household income in transforming Rural China

**DOI:** 10.1186/1471-2458-11-862

**Published:** 2011-11-12

**Authors:** Chaowei Fu, Yue Chen, Fadi Wang, Xuecai Wang, Jiangen Song, Qingwu Jiang

**Affiliations:** 1 Department of Epidemiology, School of Public Health, Fudan University; Key Laboratory of Public Health Safety, Ministry of Education, Shanghai 200032, China; 2 Department of Epidemiology and Community Medicine, University of Ottawa, Ottawa, Ontario K1H 8M5, Canada; 3 Deqing County Centre of Disease Prevention and Control, Deqing County, Zhejiang Province, 313200, China

## Abstract

**Background:**

The prevalence of hyperglycaemia and its association with socioeconomic factors have been well studied in developed countries, however, little is known about them in transforming rural China.

**Methods:**

A cross-sectional study was carried out in 4 rural communities of Deqing County located in East China in 2006-07, including 4,506 subjects aged 18 to 64 years. Fasting plasma glucose (FPG) was measured. Subjects were considered to have impaired fasting glucose (IFG) if FPG was in the range from 5.6 to 6.9 mmol/L and to have diabetes mellitus (DM) if FG was 7.0 mmol/L or above.

**Results:**

The crude prevalences of IFG and DM were 5.4% and 2.2%, respectively. The average ratio of IFG/DM was 2.5, and tended to be higher for those under the age of 35 years than older subjects. After adjustment for covariates including age (continuous), sex, BMI (continuous), smoking, alcohol drinking, and regular leisure physical activity, subjects in the high household income group had a significantly higher risk of IFG compared with the medium household income group (OR: 1.74, 95% CI: 1.11-2.72) and no significant difference in IFG was observed between the low and medium household income groups. Education and farmer occupation were not significantly associated with IFG.

**Conclusions:**

High household income was significantly associated with an increased risk of IFG. A high ratio of IFG/DM suggests a high risk of diabetes in foreseeable future in the Chinese transforming rural communities.

## 1. Background

The World Health Organization (WHO) has estimated that more than 346 million people worldwide have diabetes today, and the number will likely double by 2030 [1,2]. In China, the number of people with diabetes is expected to increase from 20.8 million in 2000 to 42.3 million in 2030 [2]. The prevalence of diabetes for all age-groups worldwide was approximately 2.8% in 2000 and will be 4.4% in 2030, with a large variation from one country to another [2,3].

During the 20th century, developed countries experienced a rapid decline in deaths from infections and childhood diseases and a rapid increase in chronic non-communicable diseases such as obesity, diabetes, and cardiovascular disease (CVD), which was associated with industrialization, mechanization, urbanization, and economic development. More recently, developing countries including China have been experiencing a similar transforming process [4-12]. Since early 1980s, mainland China has become one of these transforming societies and people have been experiencing great changes in lifestyles, living conditions, nutrition and health care, but there is a lag for people living in rural areas as compared to their urban counterparts [10-12]. High socioeconomic level indicated by income, education and occupation is generally associated with a low risk of diabetes in developed countries, although some studies has suggested no association between income or occupation and diabetes, and inverse associations were reported even in developing countries [13-20].

Along with the rapid change of economy during the past 30 years, the prevalence of type 2 diabetes mellitus (T2DM) has been increasing rapidly in urban China [21-24]; however, little is known for those living in rural communities which have been experiencing somewhat lagged changes during the period [25]. Deqing County in Zhejiang province had a net income per capita of 9773 RMB (1416 US $) in 2007 for farmers, with an increase of 14.4% compared with the previous year, and the increasing rate was more than 10% on average since 2003[26]. The study examined the current status of hyperglycaemia including impaired fasting glucose (IFG) and diabetes mellitus (DM), and explored the relationship between socioeconomic status and hyperglycaemia in adults living in rural Deqing, China.

## 2. Methods

### 2.1 Study site and population

This is the baseline survey for the Rural Deqing Cohort Study, which was conducted in four rural communities in Deqing County, Zhejiang Province. The target population included all adult residents aged 18 to 64 years, with an exclusion of those who were temporary workers or university students who were not living in the county during the period from September 2006 to May 2007.

### 2.2 Informed consent

The study was approved by the Institutional Review Board of the Fudan University School of Public Health. A written informed consent was sought after a complete description of the study narrated to each subject.

### 2.3 Subjects recruitment and data collection

Data collectors were health workers who were recruited from each study site and trained by academic investigators. They visited each household in these rural communities and identified eligible study subjects based on name lists provided by local authorities. The participants were face-to-face interviewed. If eligible subjects were not at home at the visiting, they could be face-to-face interviewed at physical examination centers. The questionnaire covered the information on individuals' demographics, lifestyles, and disease history. All subjects were asked to have a physical examination including body weight and height measuremnts. They were also asked to provide a blood sample of 5 ml next morning. Fasting plasma glucose test was performed by using glucose oxidase method within 2 hours.

Subjects were considered to have impaired fasting glucose (IFG) if fasting plasma glucose (FPG) was in the range from 5.6 to 6.9 mmol/L and to have diabetes mellitus (DM) if FPG was 7.0 mmol/L or above or they were receiving antidiabetic medications [27]. Hyperglycaemia refered to eigher IFG or DM. Farmers were those who made their living from farming at the interview and other occupations including teachers, managers, officers, staffes, workers, drivers, students, etc. were grouped into non-farmers. Household income was self-ranked by subjects as low, medium or high relative to others living in the same community. Body mass index (BMI) was calculated: BMI = weight (kg)/height^2 ^(m^2^). Smokers were those who continually smoked for more than 6 months and at least one cigarette per day. Alcohol drinking was defined as drinking any alcohol at least once a week. Regular leisure physical activity was defined as having physical activities for more than half an hour for at least twice a week. Physical activities at work or homework were not measured. Years of school education were also recorded.

### 2.4 Data analysis

All statistical tests were completed by using SPSS11.0 (SPSS Inc., Chicago, Illinois, USA; serial number: 3805233). Categorical variables were summarized by frequency count and corresponding percentage and continuous variables were summarized by mean and standard deviation. Chi-square test was used for categorical variables, and independent t test for continuous variables. In multinomial logistic regression analysis, we examined the associations of years of education, farmer occupation and household income with glycaemia status before and after adjustment for age (continuous), gender, BMI (continuous), smoking, alcohol drinking, and regular leisure physical activity. No significant interactions on a multiplicative scale were detected, and therefore, the final models included main terms only. An alpha level of ≤ 0.05 (two sides) was considered to be statistically significant.

## 3. Results

### 3.1 General characteristics

A total of 5,898 (60% of 9830 eligible study subjects) provided questionnaire information; of them 4,506 (76%) had a physical examination and provided blood specimen. Overall, 46% (42% for men and 49% for women, respectively) of the eligible population were included in the current analysis. Table 1 shows the characteristics of the participants by sex. There were more women than men with an average age of 46.1 ± 10.0 years. Majority of them received less than 9 years of school education, were farmers, believed to have medium household income, and joined the rural China cooperative medical insurance. Approximately one fifth of the participants had a BMI value of 25 kg/m^2 ^or greater. Smoking and alcohol drinking were common in men but not in women. Regular leisure physical activity was rare in both men and women.

**Table 1:** Characteristics of participants by gender from 4 rural communities, Deqing, China

Characteristics	Men (n = 1865)	Women (n = 2641)	Statistical test for gender difference
	Mean ± SD	Mean ± SD	
Age (years)	47.3 ± 9.5	46.6 ± 9.6	t = 2.56, *P *= 0.011
BMI (kg/m^2^)	22.6 ± 2.7	22.2 ± 2.9	t = 4.60, P < 0.001
	No. (%)	No. (%)	
Years of education			
≤ 9	998(53.7)	1768(67.4)	χ^2 ^= 86.02, *P *< 0.001
> 9	861(46.3)	857(32.6)	
Farmer			
Yes	1326(71.1)	1917(72.6)	χ^2 ^= 1.20, *P *= 0.274
No	539(28.9)	724(27.4)	
Household income			
Low	208(11.4)	286(11.0)	χ^2 ^= 0.15, *P *= 0.929
Medium	1487(81.5)	2121(81.9)	
High	130(7.1)	183(7.1)	
Having rural cooperative medical insurance			
Yes	1733(96.2)	2486(96.5)	χ^2 ^= 0.42, *P *= 0.515
No	69(3.8)	89(3.5)	
Regular leisure physical activity			
Yes	42(2.4)	53(2.1)	χ^2 ^= 0.26, *P *= 0.607
No	1734(97.6)	2436(97.9)	
Alcohol drinking			
Yes	853(45.7)	95(3.6)	χ^2 ^= 1168.47, *P *< 0.001
No	1012(54.3)	2546(96.4)	
Smoking			
Yes	1183(63.5)	53(2.0)	χ^2 ^= 2066.52, *P *< 0.001
No	680(36.5)	2576(98.0)	
Body mass index (kg/m^2^)			
< 25.0	1528(83.0)	2209(84.6)	χ^2 ^= 2.70, *P *= 0.225
25.0-	291(15.8)	368(14.1)	
≥ 30.0	21(1.1)	34(1.3)	

### 3.2 Hyperglycaemic prevalence

A total of 48 subjects (1.1%) reported having T2DM diagnosed by a physician and only 139 (3.1%) reported having ever been tested for blood sugars. Overall, the average level of FPG was 4.7 ± 0.9 mmol/L, ranging from1.5 to 17.1 mmol/L, with the crude prevalence of IFG and DM being 5.4% and 2.2%, respectively. The age-standardised prevalences with 2000 standard China population were 4.2% for IFG and 2.1% for DM, respectively. The crude hyperglycaemic proportion increased significantly over age from 2.8% in the 18-24 year group to 11.1% in the 55-64 year group (χ_trend_^2 ^= 43.18, p < 0.001; Table 2) with no significant difference between men and women (χ^2 ^= 3.30, p = 0.192). The average ratio of IFG/DM was 2.5 overall (5.0 for 18-34 years group, 2.4 for 35-44 years group, 2.5 for 45-54 years group and 2.3 for 55-64 years group, respectively), and was higher for those under the age of 35 years.

**Table 2:** Crude prevalence of hyperglycaemia in adults by sex and age in rural Deqing (%)

Age (years)	Male			Female		
	
	n	IFG	DM	n	IFG	DM
18-24	50(2.7)	3(6.0)	0(0)	59(2.2)	0(0)	0(0)
25-34	136(7.3)	3(2.2)	2(4.3)	254(9.6)	9(3.5)	1(0.4)
35-44	577(30.9)	20(3.5)	12(2.1)	888(33.6)	35(3.9)	11(1.2)
45-54	647(34.7)	34(5.3)	20(3.1)	857(32.4)	60(7.0)	17(2.0)
55-64	455(24.4)	30(6.6)	12(2.6)	583(22.1)	50(8.6)	23(3.9)

Total	1865(100.0)	90(4.8)	46(2.5)	2641(100.0)	154(5.8)	52(2.0)

Abbreviations: IFG = impaired fasting glucose with a fasting glucose of 5.6 to 6.9 mmol/L, DM = diabetes mellitus with a fasting glucose of ≥ 7.0 mmol/L or receiving antidiabetic medications, n = total numbers

Note: χ^2 ^= 3.30, p = 0.192 for the gender difference of hyperglycaemia.

### 3.3 Socioeconomic factors and hyperglycaemia

As shown in Table 3, subjects with more than 9 years of education had a significantly lower crude prevalence of hyperglycaemia than those with less than 9 years of education, while the high income group had a significantly higher hyperglycaemic prevalence than the medium or low income groups. After adjustment for age, sex, BMI, smoking, alcohol drinking, and regular leisure physical activity in a multinomial logistic regression model, the adjusted odds ratio for IFG was 1.74 (95% CI: 1.11, 2.72) in the high income group compared with the medium income group and the difference in IFG was not statistically significant between the low and medium income groups. After adjustment for covariates the relationship between years of education and IFG was no longer statistically significant (Table 4). There was no significant association between farmer occupation and DM.

**Table 3:** Hyperglycaemic prevalence by socioeconomic characteristics in rural Deqing

Characteristics	n (%)	IFG (%)	DM (%)	Statistical test
Education years				
≤ 9	2766(61.7)	169(6.1)	66(2.4)	χ^2 ^= 7.60, *p = *0.022
> 9	1718(38.3)	75(4.4)	31(1.8)	
Farmer				
Yes	3243(72.0)	185(5.7)	74(2.3)	χ^2 ^= 2.49, *p *= 0.288
No	1263(28.0)	59(4.7)	24(1.9)	
Household incomes				
Low	494(11.2)	20(4.0)	15(3.0)	χ^2 ^= 12.18, *p *= 0.016
Medium	3608(81.7)	191(5.3)	65(1.8)	
High	313(7.1)	27(8.6)	8(2.6)	
Total	4506(100.0)	244(5.4)	98(2.2)	

Abbreviations: IFG = impaired fasting glucose with a fasting glucose of 5.6 to 6.9 mmol/L, DM = diabetes mellitus with a fasting glucose of ≥ 7.0 mmol/L or receiving antidiabetic medications, n = total numbers

**Table 4:** Unadjusted and adjusted odds ratios (95% confidence intervals) as well as p value for socioeconomic factors associated with hyperglycaemia in rural Deqing

Hyperglycaemia	Characteristics	Unadjusted	p	Adjusted ^b^	p^b^	Adjusted ^c^	p^c^
IFG^a^	Years of education						
	≤ 9	1.44 (1.09, 1.90)	0.011	0.90 (0.65, 1.24)	0.517	0.99 (0.70, 1.40)	0.961
	> 9	1.00		1.00			1.00
	Farmer						
	No	1.23 (0.92, 1.67)	0.161	1.08 (0.79, 1.49)	0.620	1.03 (0.74, 1.43)	0.876
	Yes	1.00		1.00			1.00
	Household income						
	Low	0.67 (0.41, 1.12)	0.124	0.63 (0.38, 1.05)	0.077	0.74 (0.45, 1.21)	0.229
	Medium	1.00		1.00			1.00
	High	1.78 (1.17, 2.72)	0.007	1.93 (1.26, 2.96)	0.002	1.74 (1.11, 2.72)	0.015
DM^a^	Years of education						
	≤ 9	1.36 (0.88, 2.09)	0.166	0.74 (0.44, 1.25)	0.261	0.93 (0.54, 1.59)	0.787
	> 9	1.00		1.00			1.00
	Farmer						
	No	0.82 (0.51, 1.31)	0.404	1.18 (0.71, 1.97)	0.526	1.10 (0.66, 1.84)	0.723
	Yes	1.00		1.00			1.00
	Household income						
	Low	1.69 (0.95, 2.98)	0.073	1.59 (0.90, 2.83)	0.111	1.79 (0.98, 3.27)	0.058
	Medium	1.00		1.00			1.00
	High	1.48 (0.71, 3.12)	0.299	1.66 (0.78, 2.51)	0.186	1.55 (0.72, 3.34)	0.261

Abbreviations: IFG = impaired fasting glucose with a fasting glucose of 5.6 to 6.9 mmol/L, DM = diabetes mellitus with a fasting glucose of ≥ 7.0 mmol/L or receiving antidiabetic medications, n = total numbers

Note: ^a ^Normal FPG as the reference category.

^b ^Adjusted for age (continuous) and sex.

^c ^Adjusted for age (continuous), sex, BMI (continuous), smoking, alcohol drinking, and regular leisure physical activity.

## 4. Discussion

The data from the Chinese National Diabetes Survey showed that the prevalence of diabetes increased from 1.0% in 1980 to 2.5% in 1994 [21,22]. Data from a representative national survey in 2002 demonstrated that for T2DM the age-standardized prevalence was 4.5% in urban China and 1.8% in rural China, and for IFG they were 2.7% and 1.6% [23]. The age-standardized prevalences of IFG (4.2%) and DM (2.1%) in rural Deqing in our study were comparable with those for urban China in 2002 [23] and those for rural Tianjin in 2004 [25]. Overweight and obesity is a major determinant for diabetes [28]. The prevalence of overweight/obesity and mean BMI in rural Deqing was comparable to the national average in the country [23,24].

IFG or impaired glucose tolerance is associated with an increased risk of diabetes [29-35]. It has been suggested that the ratio of IGT prevalence versus diabetes mellitus prevalence may a useful indicator for future risk of type 2 diabetes [36,37]. The average ratio of IFG/DM was markedly higher in this study compared with another study conducted in 2002 [23], and tended to be higher for those under the age of 35 years than older subjects, which may suggest a rapider increase in diabetes risk among the younger population in foreseeable future.

In developing country, adults with higher income have a higher risk of chronic diseases including diabetes, partly due to their earlier westernized transition of lifestyle and diet [10-12,19,20]. In China, a study reported that rapid income growth adversely affected Chinese diet quality [38]. This study provided a glimpse of current status of hyperglycaemia in those living in one richer rural region in China. High household income seemed to be a risk factor for IFG during a transforming process in rural China, which is consistent with some previous observations from developing nations [19,20]. High household income was also associated with an increasing risk of DM but this association was not statistically significant. In addition, although not significant, subjects with low household income had a lower risk of IFG (aOR = 0.74), but had a higher risk of DM (aOR = 1.74), compared with those with medium household income. Lack of statistical power may be one of possible reasons for these observations, and further studies are needed for any confirmation. After adjustment for covariates, education was no longer associated with hyperglycaemia. In rural China, people spend a large proportion of their income for food and some types of food such as meat products are more affordable for relatively wealthy people, which may explain our observation that high income but not high education was a risk factor for IFG.

There are some limitations for the study. This is a cross-sectional study and the evidence is weak for a possible causal linkage although it is not likely that poorer status of hyperglycaemia would result in higher household income. Another limitation is that less than 50% of the eligible subjects provided blood specimens. If people with perceived poor health were more likely to participate in the study, we might overestimate the hyperglycaemic prevalence. In this study, individuals who provided blood specimens, compared with participants who did not provide blood specimens, were older, less educated and heavier, and were more likely to be female and to have high blood pressure (diastolic blood pressure ≥ 90 mmHg and/or systolic blood pressure ≥ 140 mmHg or receiving antihypertensive medications). However, there were no differences in household income and regular leisure physical activity. Thirdly, FPG test instead of oral glucose tolerance test (OGTT) may underestimate the prevalence of diabetes. A fasting plasma glucose cut-off of 5.6 mmol/L, however, has been found to be comparable to an OGTT cut-off of 7.8 mmol/L [27,39]. Fourthly, we used household income, which was self-ranked by the participants. People were less likely to report their income in Yuan value (80%) compared with relative household income (98%). There was an impression the people tended to under-report their income in Yuan value while a question on relative household income was less sensitive. In this study, self-ranked relative household income was significantly but not highly correlated to average household income per capital among subjects who provided both (Spearman's correlation coefficient = 0.44, p < 0.001). Similar trends were observed when income was measured in Yuan values, but the estimates were less precise due to much smaller sample size. Fifthly, regular leisure physical activity was not common for this rural population and was not related to hyperglycaemia. Work related physical activity may be more relevant for rural people in China, which was not measured in this study. Lastly, there may be a household cluster effect but the information was not collected in this study.

## 5. Conclusion

In conclusion, the hyperglycaemic prevalence for adults living in a richer rural area was comparable to the average of Chinese urban population. An elevated ratio of IFG/DM suggests a high risk of diabetes in foreseeable future in the Chinese transforming rural communities. High household income was a risk factor for the prevalence of IFG. Intervention strategies should be developed for rural residents during the process of the rapid socioeconomic development.

## 6. Competing interests

The authors declare that they have no competing interests.

## 7. Authors' contributions

CF carried out this survey, participated in its design, performed the statistical analysis, and drafted the manuscript. YC participated in study design and was involved in revising the manuscript critically for important intellectual content. FW participated in the coordination and study design. XW participated in the coordination and data collection. JS participated in the coordination and data collection. QJ participated in study design and revised it critically for important intellectual content. All authors read and approved the final manuscript.

## 8. Authors information

Jiang, Qingwu MD, MPH

Professor of Epidemiology

138 Yi Xue Yuan Road

Department of Epidemiology

School of Public Health

Fudan University

Shanghai 200032, China

Tel: 86 21 54237435

Fax: 86 21 54237435

Email: jiangqw@fudan.edu.cn

## Pre-publication history

The pre-publication history for this paper can be accessed here:

http://www.biomedcentral.com/1471-2458/11/862/prepub
